# Complete genome sequence of *Marinomonas* sp. strain THO17 isolated from the leaf surface of the seagrass (*Thalassia hemrichii*)

**DOI:** 10.1128/mra.00723-24

**Published:** 2024-11-13

**Authors:** Yuki Tsuchiya, Ushio Yokoyama, Reiji Takahashi, Tatsunori Nakagawa

**Affiliations:** 1 Department of Bioscience, College of Bioresource Sciences, Nihon University, Fujisawa, Kanagawa, Japan; 2 Graduate school of Bioresource Sciences, Nihon University, Fujisawa, Kanagawa, Japan; California State University San Marcos, San Marcos, California, USA

**Keywords:** *Marinomonas*, seagrass, plant–microbe interactions

## Abstract

Here, we report the genome sequence of the *Marinomonas* sp. strain THO17. The assembled genome consisted of a single circular chromosome of 3,920,899 bp, with a 43.6% G+C content. Genome analysis revealed that strain THO17 potentially interacts with marine plants.

## ANNOUNCEMENT

Several members of *Marinomonas* have been detected and isolated from marine plants such as seagrasses and eelgrasses (1–4) and have been suggested to play an important role in enhancing the growth of marine plants (5).

The *Marinomonas* sp. strain THO17 was isolated from the leaf surface of the seagrass (*Thalassia hemrichii*) cultivated in the 10 L aquarium at 25°C under the light–dark (12 hour:12 hour) cycle. Bacteria attached to the leaves were removed using a sterilized sponge and suspended in sterilized seawater. The suspension was inoculated on marine agar using the serial dilution method and was incubated at 25°C. After 2 weeks, a single colony, designated THO17, was selected and isolated using the streak plate method. THO17 cells were cultured in a marine broth for sequencing (25°C, 7 days).

The genomic DNA of THO17 was extracted using the NucleoBond Buffer Set III and an AXG 500 column (TaKaRa Bio, Shiga, Japan), following the manufacturer’s instructions. The extracted DNA was sheared into 10–20 kbp fragments using g-TUBE (Covaris, MA, USA) and used to generate the library using the SMRTbell Express Template Prep Kit 2.0 (PacBio, CA, USA), following the manufacturer’s instructions. The library was used to form polymerase complexes using Binding Kit 2.2 (PacBio). The complexes were sequenced using a Sequel IIe system. After sequencing, the adapter sequence was deleted, and 20,517 high-fidelity (HiFi) reads (average 12,085 bp) with quality values higher than 20 were obtained using SMRT Link (v.11.0.0.146107). HiFi reads exceeding 1 kbp obtained from Filtlong (v.0.2.1) were *de novo-*assembled and circularized using Flye (v.2.9.1-b1780) (6) run with default parameter and no reference, resulting in a single circular genome sequence of 63.2 × coverage and N50 length of 3,920,899  bp with a 43.6% G + C content. An assembly graph was visualized by Bandage v.0.8.1 (7) with default parameters. Genome completeness and contamination were estimated using CheckM v.1.2.2 (8), resulting in 99.9% and 0.69%, respectively. The genome sequence was annotated using the programs implemented in the DFAST v.1.3.0 pipeline (9) using default parameters. In total, 3,600 protein-encoding genes, 24 rRNA genes, and 76 tRNA genes were identified.

The 16S rRNA gene of THO17 was compared with the gene of other *Marinomonas* representatives deposited in the NCBI 16S rRNA database v1.1 using the BLASTn program v2.16.0 (10). THO17 showed the greatest similarity (97.1%) with *Marinomonas aquiplantarum* strain IVIA-Po-159 (GenBank accession number NR116236). The average nucleotide identity (ANI) between THO17 and *M. aquiplantarum* IVIA-Po-159 was estimated to be 81.6% using FastANI v.1.33 (11). Further taxonomic analyses are necessary to clarify the species of THO17.

Genes putatively involved in plant–microbe interactions (12), such as genes related to iron–siderophore complexes (13), inorganic phosphorus transporters (14), and indole-3-acetic acid (15), were predicted in the chromosome of THO17.

## Data Availability

The genome sequence of *Marinomonasa* sp. THO17 has been deposited in DDBJ under accession number AP031575 (BioProject number PRJDB18076, GenBank Assembly accession number GCA_040436405.1GCA_040436405.1) and in the DDBJ Sequence Read Archive (DRA) under accession number DRR567738. The output of DFAST is available on Figshare (https://doi.org/10.6084/m9.figshare.27201036.v2).
